# Retrospective clinical and X-ray-based outcome analysis of a short-stem hip arthroplasty taking into account the operative learning curve over 7 years in the 3-year control course

**DOI:** 10.1007/s00402-023-04977-w

**Published:** 2023-07-07

**Authors:** Alexander Jahnke, Jan Köther-Herrmann, Carlos A. Fonseca Ulloa, Torben Harz, Markus Rickert, Bernd Alexander Ishaque

**Affiliations:** 1 Laboratory of Biomechanics, Justus-Liebig-University Giessen, Klinikstrasse 29, 35392 Giessen, Germany; 2 Department of Orthopaedics and Orthopaedic Surgery, University Hospital Giessen and Marburg (UKGM), Klinikstrasse 33, 35392 Giessen, Germany

**Keywords:** Learning curve, Short stem, THA, Follow-up, Fit&fill

## Abstract

**Introduction:**

Self-monitoring is crucial to work progressively with a high-quality standard. A retrospective analysis is a valuable tool for studying the postoperative outcome of a prosthesis and for evaluating the learning process for the surgeon.

**Materials and methods:**

The learning process of one surgeon was analysed in 133 cases of hip arthroplasty. These were divided into seven groups representing the surgical years 2008–2014. Over the course of 3 postoperative years, a total of 655 radiographs were analysed at regarding three radiological quality parameters (centrum-collum-diaphyseal angle (CCD angle), intramedullary fit&fill ratio (FFR), and migration) and ancillary outcome parameters (Harris Hip Score (HHS), blood loss, operating time, and complications). This period was divided into five times: 1st-day post-op, 6 M, 12 M, 24 M, and 36 M. Bivariate Spearman's correlation analysis and pairwise comparisons were performed.

**Results:**

The total collective achieved a proximal FFR of over 0.8. The distal prosthesis tip migrated and was located on the lateral cortex within the first months. The CCD angle initially showed a variation with a subsequent constant course. The HHS showed a significant increase (*p* < 0.001) to over 90 points postoperatively. Over time, the operating time and blood loss decreased. Intraoperative complications existed only at the beginning of the learning phase. A learning curve effect can be determined for almost all parameters by comparing the subject groups.

**Conclusions:**

Operative expertise was shown to gain through a learning curve, whereby postoperative results corresponded to the system philosophy of the short hip stem prosthesis. The distal FFR and the distal lateral distance could represent the principle of the prosthesis, which overall could be an interesting approach for verification of a new parameter.

## Introduction

Implantation of cementless hip prostheses with standard stems plays an important role in today's arthroplasty [1]. Cementless stems lead to a permanent change and displacement of the force transmission from prosthesis to bone. Due to different moduli of elasticity of the bone and the prosthesis, there is usually a reduced load in the proximal femur and consequently bone atrophy [2]. This phenomenon leads to an increase of relative movements in the periprosthetic bone via bone substance losses in the trochanteric region, resulting in losses of stability [3, 4]. Orthopaedics and orthopaedic surgery have the goal of developing primary endoprosthetic care that is as gentle on bone and soft tissue and allows for prosthesis replacement that is as atraumatic as possible. The answer to this objective is found in the short-stem prosthesis. Its implantation allows partial preservation of the collum femoris, which also serves as an anchor. Accordingly, the load acting on the prosthesis would be distributed to the intertrochanteric region via medial portions of the femoral neck cortex and along the lateral diaphysis cortex. Overall, the physiological baseline should be better matched and bone atrophy should be minimized or avoided [5]. The implantation of a short-stem prosthesis is more demanding to that of a standard stem system and generally has a lower error tolerance as short stems should be implanted as large as possible and with cortical anchorage to achieve maximum fit&fill [6]. However, the choice of a maximum dimensioned short stem can also lead to intraoperative periprosthetic fractures more quickly. The aim of this study was to determine whether the operative expertise of a surgeon improves when using the Metha^®^ short-stem prosthesis over 7 years, and how the postoperative outcome of the patient groups differ according to the date of surgery.

## Material and methods

Several radiological factors were determined as quality parameters of the postoperative outcome over a follow-up of 3 years: The intramedullary fit&fill ratio (FFR), subsidence (S), and the centrum-collum-diaphyseal angle (CCD angle). Between January 2007 and June 2020, 620 patients were treated with a Metha® short stem at the JLU Giessen Department of Orthopaedics and Orthopaedic Surgery. In the observed period from March 2008 to September 2014, 133 patients from this population who had undergone surgery by one surgeon (senior author) and had additionally appeared for all five postoperative radiological and clinical follow-up visits could be included in this study. Exclusion would occur if consent was cancelled, follow-up appointments were not kept, or patients died during the study period. During the three-year follow-up, data collection was performed on all patients at five follow-up appointments. Pelvic overview images were obtained in anterior–posterior (a.p.) and were retrospectively measured and analysed. A positive ethics vote is available (file number: 209/18).

### Target parameters

In addition to the main outcome parameters of FFR, S, and CCD angle, patients were clinically assessed at follow-up controls. The follow-ups took place immediately postoperatively (post-op), after 6 months (6 M), 12 months (12 M), 24 months (24 M), and 36 months (36 M). The patient population was divided into seven groups (1–7) regarding the patients' respective dates of surgery. Demographic factors, such as gender, age, height, weight, and BMI of the subjects, as well as clinical parameters, such as Harris Hip Score (HHS), operative time, intraoperative blood loss, and intraoperative complications, were collected and analysed as secondary outcome parameters.

### Radiological evaluation

The measurement parameters FFR, S, and CCD angle were collected in a standardized manner. All radiographs were calibrated using a magnification factor of 1.15. To avoid rotation errors, the patients were placed in a supine position and fixed in a neutral zero position of the hip thanks to positioning aids. A planning sphere (diameter 25 mm) was used to correct the magnification factor. All radiographs were analysed using the software mediCAD (Hectec, Landshut, Germany; version 4.0.0.7). To ensure that all measurements were oriented to the same anatomical landmarks and that individual deviations were as small as possible, the femoral shaft axis (FSA) was determined for all radiographs, to which all measurements were oriented.

### Stem migration

Possible migration of the prosthesis was determined by the change in distance (S) between the prosthetic shoulder and the cranial tip of the greater trochanter. The distance (*l*) was determined both immediately postoperatively and at the respective follow-ups. With a measurement inaccuracy of 1 mm of the software, the following spoke displayed a clinically relevant migration from a value of at least 3 mm.

### Fit&fill ratio

**Fig. 1:**
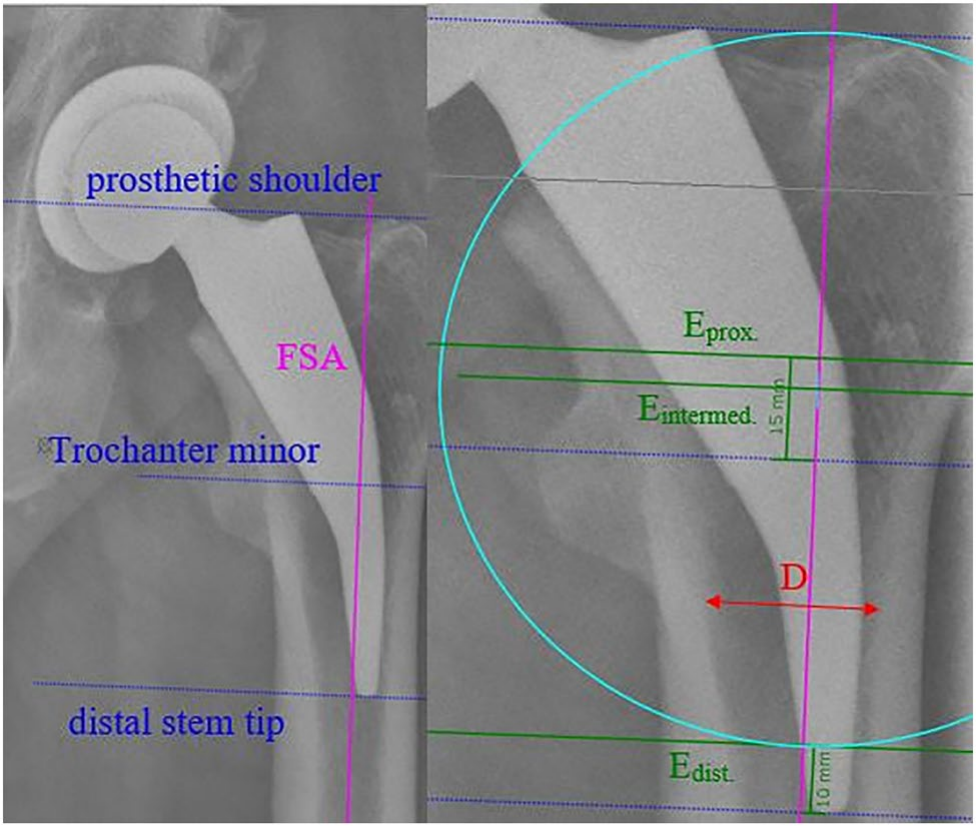
Determination of the FSA and the measurement levels

FFR was determined at three different positions (FFR_proximal_, FFR_intermediate_, and FFR_distal_). To calculate the FFR, the FSA was first determined. The reference points (prosthetic shoulder, lesser trochanter, and prosthetic stem tip) were marked using three orthogonal and standardized planes (E_proximal_, E_intermediate_, and E_distal_) to the FSA. Thus, E_proximal_ was 15 mm above the tip of the trochanter minor, E_distal_ was 10 mm proximal to the tip of the stem, and E_intermediate_ was midway between E_distal_ and the prosthetic shoulder (Fig. 1).

**Fig. 2:**
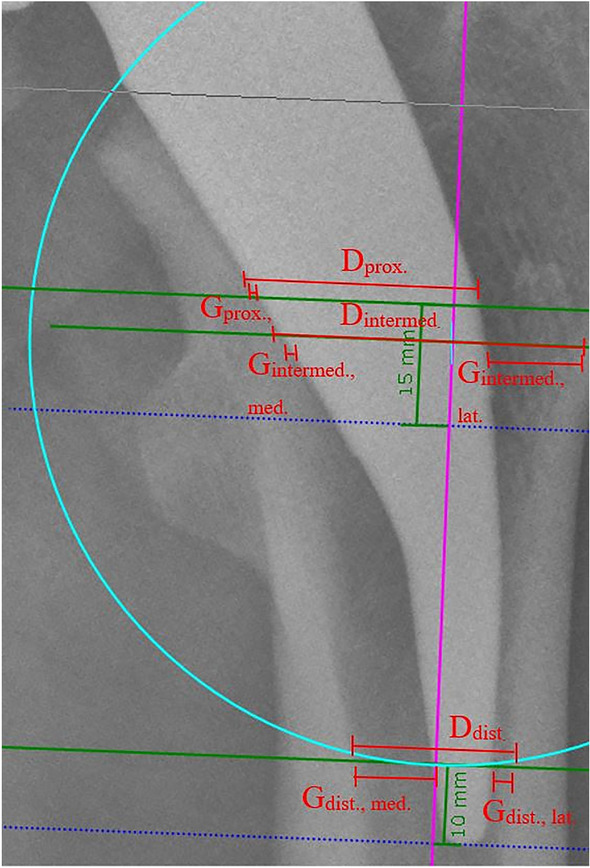
FFR determination at all levels

To determine the FFR, the distance (D) between the inner lateral and the inner medial cortex on all three measurement planes was measured. In addition, the medial gap (*G*_med_.) and the lateral gap (G_lat_.) were determined. In the proximal measurements, the lateral gap could be neglected because a clear identification of the lateral cortex was not possible. Thus, the distance in this area could only be defined as the distance between the inner medial cortex and the lateral edge of the prosthesis. The Metha^®^ short-stem prosthesis aims for metaphyseal anchorage and apposition along the lateral cortex in the distal region. Accordingly, not only the distal FFR but also the distal lateral gap between the prosthesis and the lateral cortex were evaluated (Fig. 2).

Accordingly, the FFR results from the following formula:

$${\text{FFR}} = \frac{{D - \left( {G_{{{\text{lat}}}} + G_{{{\text{med}}}} } \right)}}{D}.$$

A quotient ≥ 0.8 indicates that the prosthesis fills ≥ 80% of the site and is interpreted as stable, and values < 0.8 as not stable [6, 7].

### Statistics

Statistical analysis was performed using SPSS version 26 (SPSS Inc. Chicago IL). Normal distribution was examined using the Kolmogorov–Smirnov test and the Shapiro–Wilk test. Where a non-normally distributed collective was present, the Kruskal–Wallis test and a Spearman bivariate correlation analysis were applied for the metric-scaled variables.

## Results

### Collective

**Fig. 3:**
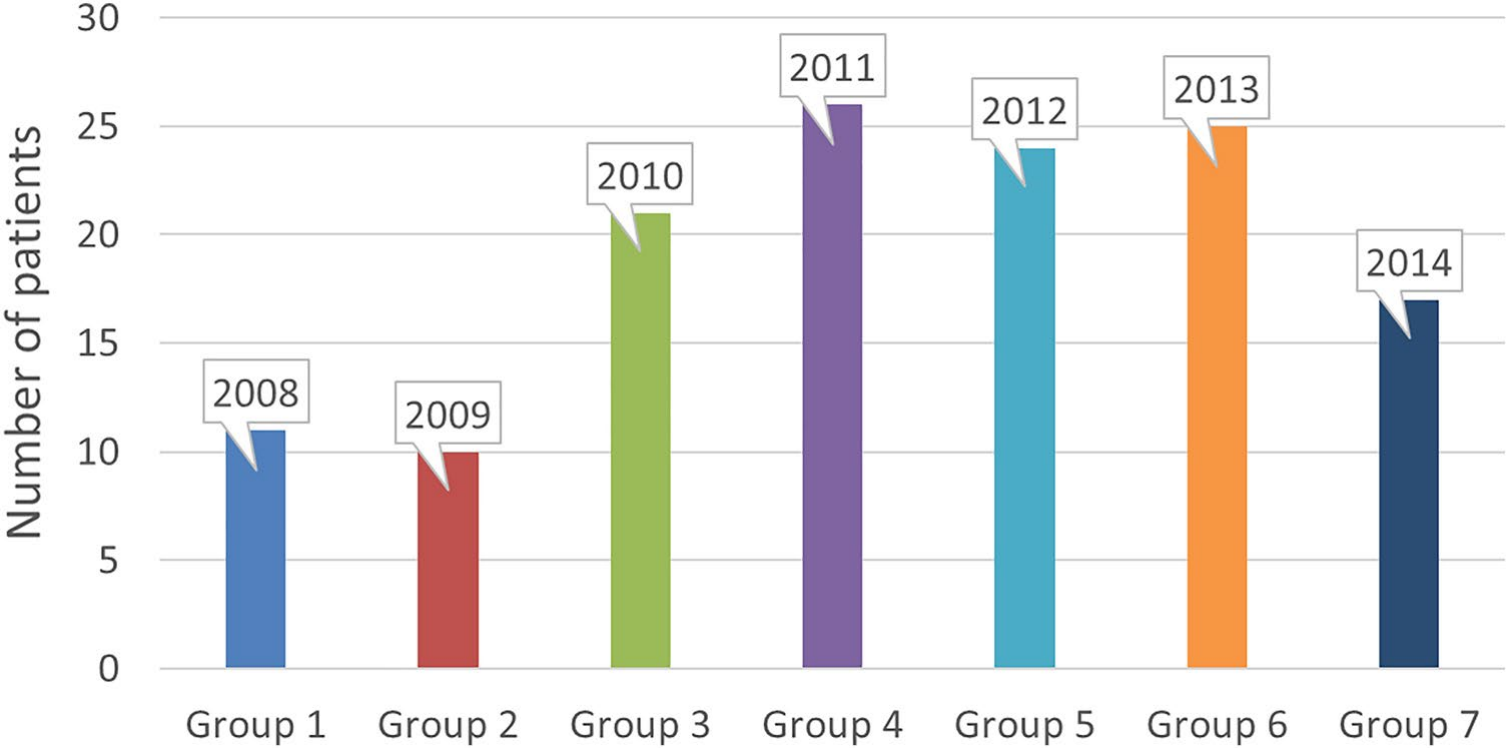
Patient distribution within the groups

The collective consisted of 70 women (53%) and 63 men (47%). Sixty-four operations (48.1%) were performed on the right and 69 implantations (51.8%) on the left hip joint. The most surgical indication was primary coxarthrosis with 83 cases (62.4%), followed by dysplasia coxarthrosis with 32 (24.1%) and femoral head necrosis with 13 cases (9.8%). Other indications included coxarthrosis due to post-trauma, and other previous diseases or surgeries, with a total of five cases (3.8%). Most patients were distributed almost equally among groups 3 to 6 (72% in total). The groups of 2008 and 2009 contained the lowest proportion of subjects (15% in total) (Fig. 3).

The mean age of the total collective was 55 years, with a standard deviation of ± 12.1 years (range 17–78). Body mass index (BMI) averaged 27.4 kg/m^2^, with a standard deviation of 4.9 kg/m^2^.

### Centrum-collum-diaphyseal angle

**Fig. 4:**
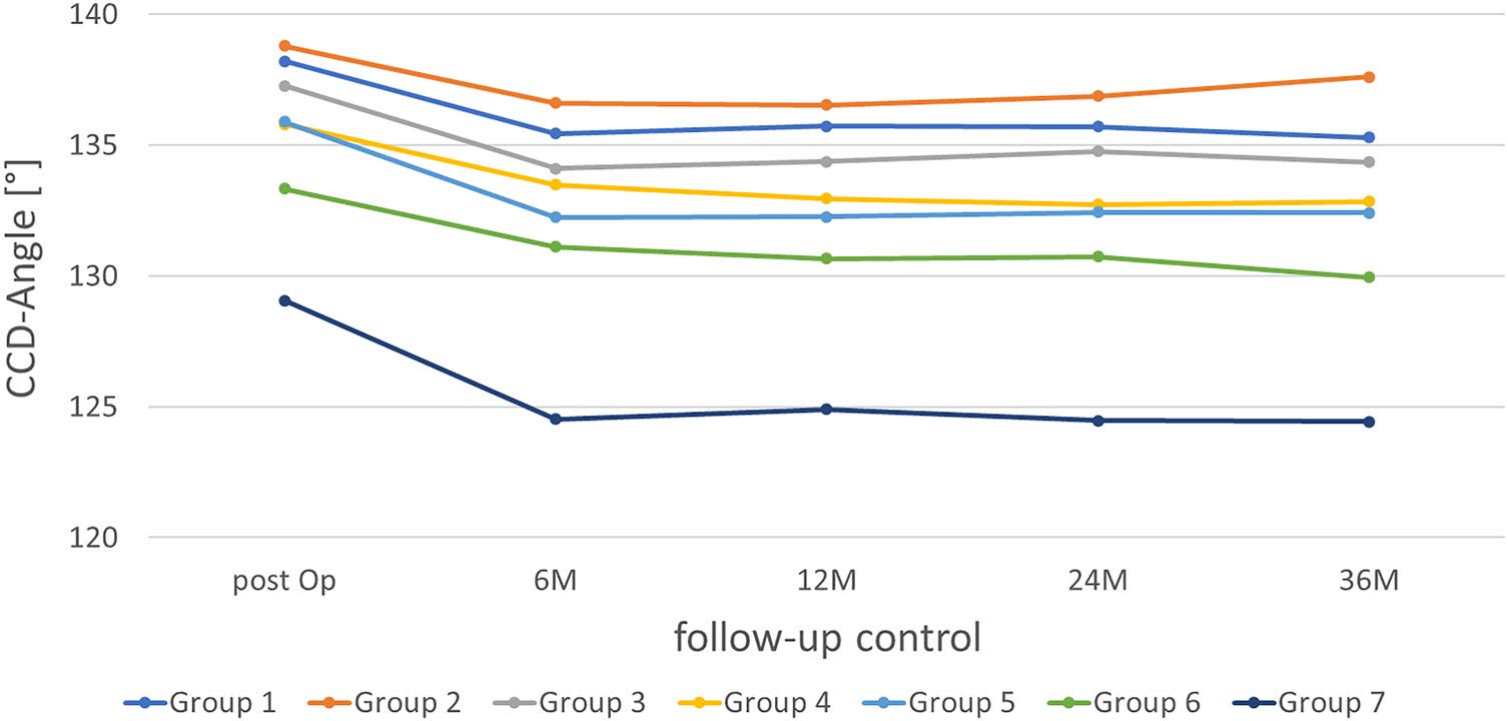
Changes in CCD angle of all groups in the follow-up period

The CCD angle in the whole collective on postoperative day 1 was on average 135.2° ± 6.7°. A reduction of the CCD angle average from post-op to 6 M could be observed. Subsequently, the angle stagnated over the listed 3 years. This trend was also reflected when looking at the CCD angle as a function of the follow-up dates of the individual group-specific data. All groups had a lower CCD angle at 6 M than at post-op and, for the most part, stagnated consistently over time. In contrast, group 2 showed a minimal increase in CCD angle after 36 M and group 3 after 24 M (Fig. 4).

### Fit&fill ratio

**Fig. 5:**
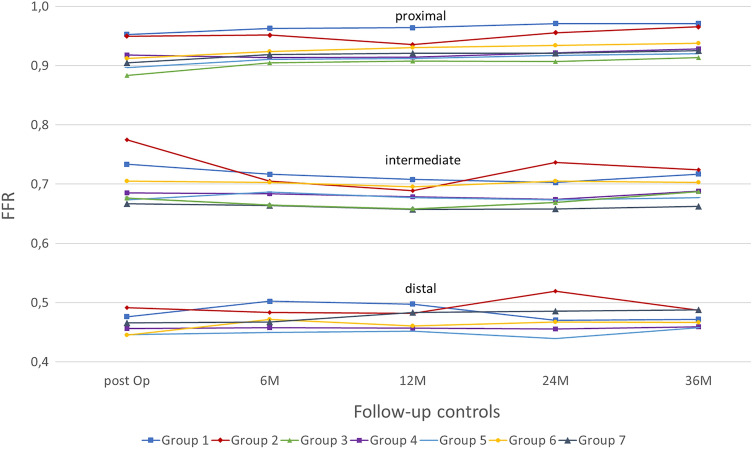
Course of the FFR of the individual groups

The proximal FFR was on average 0.91 ± 0.05 and increased to a value of 0.93 ± 0.05 at 36 M. A positive slope was also found in the progression of the group-specific graphs. Except for groups 2 and 4, the other ones showed a steady positive trend. Group 2 recorded a minimum (0.94) at 12 M, and group 4 recorded a minimum (0.91) at 6 M and 12 M. The intermediate FFR averaged 0.69 ± 0.08 at post-op, reached its minimum after 12 M (0.68 ± 0.08), and ended with a value of 0.69 ± 0.08 after 36 M. The overall intermediate FFR also showed a constant trend among the groups. Except for group 2, the post-op measured medullary gap corresponded to the measured value obtained after 36 M. Comparing groups, groups 1 and 2 recorded slightly higher measured values than the others. An average value of ≥ 0.8 was not achieved by any of the curves. The distal FFR averaged at 0.46 ± 0.07. This value changed marginally over time, reaching 0.47 ± 0.08 after 36 M. In comparison to the other ones, group 3 had on average significantly higher FFR values of 0.66 to 0.69, whereas the other groups achieved a value range of 0.45 to a maximum of 0.52 (Fig. 5).

**Fig. 6:**
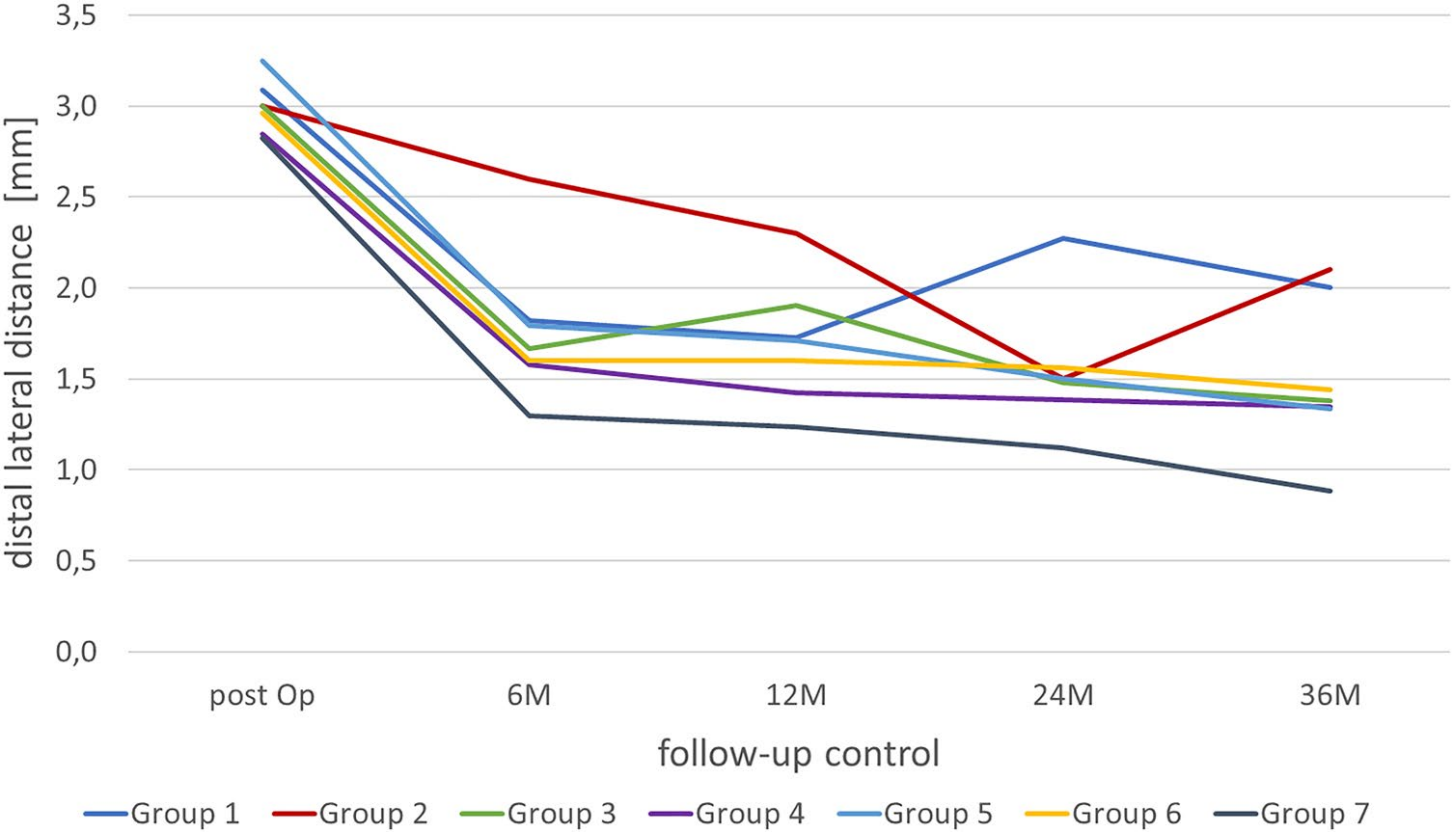
Course of the distal lateral distance of the individual groups

Overall, a downward trend could be seen during the three-year period. In addition, groups 1 to 3 showed a brief increase in the distal lateral gap, which, however, never exceeded the initial value. All groups showed a range of values from 2.8 to 3.1 mm and ended at a minimum of 0.88 and a maximum of 2.1 mm. It was evident that as the years of surgery increased, the initial postoperative distal lateral distance decreased and the values and final values decreased significantly more as the year of surgery progressed (Fig. 6).

### Migration

**Fig. 7:**
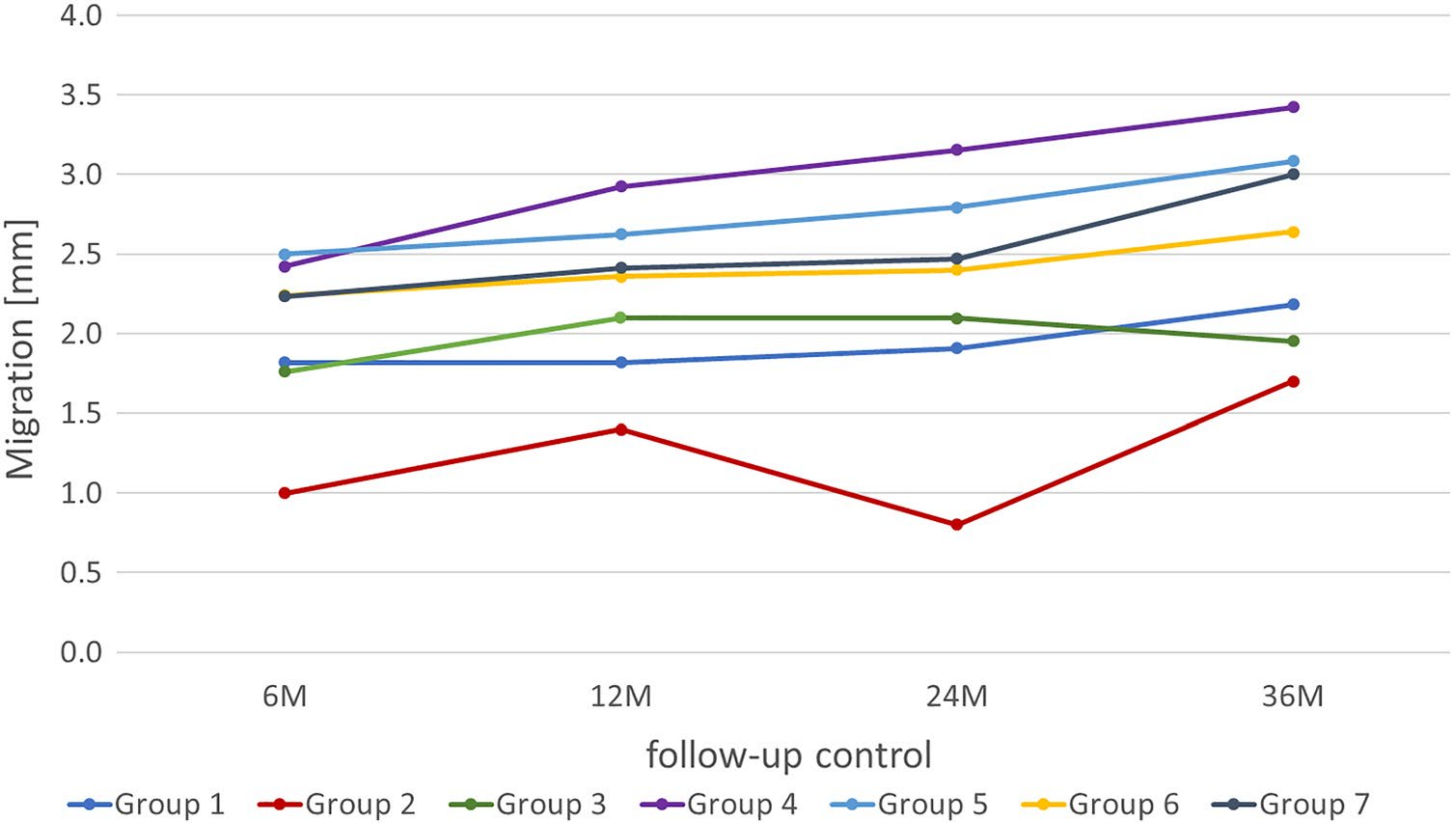
Migration course of the individual groups

The collective recorded values from 0 mm to a maximum of 12 mm for migration over the total period. The mean of the collective ranged from 2.1 ± 1.8 mm from post-op to 2.7 ± 2.1 mm to 36 M. The upward trend, which was already evident in the increasing mean values of the overall collective, also applied to the individual groups. Groups 1 to 3 showed the lowest migration values (1–2.18 mm), followed in ascending order by groups 6, 7, 5, and 4. Only the last two graphs of the groups exceeded the limit of 3 mm (Fig. 7).

### Clinical parameters

The total operative time was 127 min for group 1 and 119 min for group 2. The maximum was reached in group 3 with 128 min. In the further course, the op-time decreased and reached a minimum of 94 and 95 min in groups 6 and 7. Overall, the op-time decreased by approximately 32 min from the beginning of the incision to the end of the suture from groups 1 to 7. The average intraoperative blood loss of the respective groups 1–7 was 585 ml, reaching the average in group 1 and increasing to 690 ml by group 3 and decreasing thereafter. In the process, blood loss in group 4 returned to the baseline value of group 1 and fell below it to the nadir in group 7 at 490 ml. The pairwise comparison revealed a significant increase in HHS (*p* < 0.001) from preoperative to all postoperative values. There was no significant change in HHS between groups at the respective follow-up date.

### Correlation of the measurement parameters

No significant correlations could be demonstrated among the FFR, the CCD angle, and the migration values over the observation period. There were also no correlations between the epidemiological factors and the radiological values.

## Discussion

In this study, 665 anterior–posterior pelvic images of a total of 133 patients between the years 2008–2014 were retrospectively analysed by one investigator regarding three radiological quality parameters on five defined control dates. Compared with other studies, which analysed collective sizes ranging from a maximum of 22 up to 250 cases, this study can be ranked in the upper middle range in terms of sample number [7–19]. Only subjects who attended all 5 follow-up appointments were included. Thus, the collective size remained constant over the entire observation period and allowed for high reliability. Endoprosthetic treatment was always performed by the same surgeon and according to a standardized procedure. Surgical experience, and thus implant size decision patterns, is subject to a learning process [20, 21].

### Centrum-collum-diaphyseal angle

The initial hypothesis dealt with the question of whether the CCD angle of the prosthesis decreases in the postoperative course. The results showed that after an initial decrease within the first 6 months, the averaged CCD angle was constant. In summary, the first hypothesis was confirmed.

A similar result was also reached by the Jerosch et al., who described that certain metadiaphyseal fixed prosthesis models tend to a system-related valgization [22].

Kutzner et al. investigated the biomechanical influence of the CCD angle using the metaphyseal anchored Optimys^®^ short-stem prosthesis (Mathys Ltd., Bettlach, Switzerland) in a postoperative interval of 2 years. They found that valgus alignment leads to increased initial migration, but the clinical outcomes remain unaffected [23, 24]. Moreover, no significant correlation was found between the CCD angle and the process of “stress shielding” or cortical hypertrophy. Accordingly, a varus-emphasized positioning would allow the tip of the prosthesis to be supported along the lateral cortex, as is desired for metaphyseal short-stem prostheses. In valgus alignment, as well as if the stem sizes were chosen too small, this contact would often be absent. Luger et al. found that varus stem alignment greater than 3° increased hip offset and led to the risk of undersizing the prosthesis [25]. Another investigation showed that there was no difference in early fracture or failure rates between varus and neutrally aligned stems. Forced intraoperative correction of mild varus stem alignment may not be necessary and would unnecessarily increase fracture risk [26]. Another study investigated a short- to medium-term migration analysis of the Metha^®^ prosthesis using EBRA-FCA [27]. Notable varus and valgus tilt were observed within the first 3 months. Especially for the Metha® prosthesis, a valgus position should be avoided, which could lead to migration without proximal-lateral support [28]. Neither the present work nor the previously mentioned studies did the CCD angle continue to increase during the course after the initial increase. Accordingly, the initial change could be interpreted as the “settling” of the prosthesis and would depend solely on the initial positioning. A change in position would therefore be considered a sign of loosening for the Metha® prosthesis if it occurs after the initial phase of the first 6 to 12 months of “settling” [15].

### Fit&fill ratio

Distal FFR averaged at 0.47. The intermediate FFR reached a mean value of 0.69, in contrast to the proximal FFR, which increased from 0.91 to 0.93. Likewise, the relative proportion of patients with a proximal FFR value of ≥ 0.8 or ≥ 0.9 increased. On the first postoperative day, 130 of 133 cases showed an intramedullary seat near or above 0.8, which increased by two more cases after 3 years. Whereupon, the preoperative outcome secured proximal primary stability, and consequently, optimal secondary stability was achieved. Jahnke et al. analysed the influence of the marrow space fit of the Metha^®^ prosthesis 3, 6, and 12 months postoperatively. They achieved a proximal intramedullary canal fit of ≥ 0.8 in 100% of patients, and a sufficient intramedullary canal fit was observed for the intermediate FFR in 67.5% (3 M), 56.4% (6 M), and 42.6% (12 M) of cases. In the distal measurement area, the proportion of FFR ≥ 0.8 was 25.0% (3 M), 23.1% (6 M), and 10.3% (12 M), respectively [6]. This illustrates that the values of the intramedullary fit of the present study were able to meet the preoperative expectations for the implant and offer valuable results regarding the postoperative radiological outcome [13, 29, 30].

According to the anchorage strategy of the short-stem prosthesis, both a high metaphyseal fit and support of the distal tip of the implant along the lateral cortex should be aimed for [19, 31]. Accordingly, an intramedullary fit of < 0.8 would have to be achieved in the distal section. As a relative measure, the FFR cannot represent the process of distal apposition. In the present study, however, the distance between the lateral cortex and the prosthesis was also determined for the first time. This distal lateral distance can thus map the attachment process of the prosthesis and could be evaluated in the future as a new radiological parameter for evaluating the fit of a short-stem prosthesis.

In this context, FFR measurements reflect the targeted force transmission of the prosthesis through the intertrochanteric region, the medial portions of the femoral neck cortex, and the lateral shaft cortex. Bone density measurement can reflect force transmission by detecting bone remodelling processes [32].

### Migration

Outcome analysis of migration showed an average distance under 3 mm, which implies no clinical relevance at a cut-off value of ≥ 3 mm. However, when the groups are considered individually, it was shown that the majority of migrations occurred within the first 6 months. Progressive or excessive migrations occurred only in isolated cases. Nevertheless, despite some migration values of over 3 mm and once up to 12 mm, no aseptic loosening requiring revision surgery was realized within the observation period of 36 months. Kutzner et al. mentioned axial migration of over 1.5 mm in 39% of cases after 24 months. Like the present study, they described a steady reduction of migration during the examination, which was considered stable after 2 years [33]. Jahnke et al. found comparable results for the Metha® prosthesis, that migration initially reached its maximum, and it remained constant over time [6]. De Waard et al. also observed secondary stabilization after initial migration, which indicated a minor risk of long-term aseptic loosening [34]. Ries et al. described that the migration process occurred within the first weeks and months after full loading, as well as the osseointegration, which usually occurred within the 4th- to 12th-week post-op [35, 36]. In summary, the occurrence of migration within the first 6 postoperative months is not synonymous with implant loosening, but is an expression of bone associated remodelling processes.

### Learning curve

Over the period from 2008 to 2014, a comparison of the groups revealed a learning curve effect for almost all defined parameters. According to current knowledge, this work on short-stem prosthetics is the first to track the learning success of a surgeon over 7 years and quantify it using radiological and clinical measurements. The fact that surgical experience and thus the decision patterns for implant size are subject to a learning process is also confirmed in other studies [20, 21]. Both the total operating time and the pure incision–suture time decrease with increasing operating year. This time saving may be due to the increasing experience of the surgeon and to the gaining knowledge of the entire surgical team in using the then newly prosthetic system. Cheng et al. maintained that prolonged operating time increases the risk of infection at the surgical site [37]. Another aspect that has a negative impact on postoperative outcomes is intraoperative blood loss. In the present study, the average maximum blood loss of all groups was 690 ml, and intraoperative blood transfusion was necessary in only one case in group 5 and two cases in group 6. When comparing groups 1–7, there is a decrease in intraoperative blood loss, which could be considered as a learning curve effect. Already 6 months postoperatively, all groups had an HHS score > 90 points. The improvement in HHS from pre- to postoperative was found to be statistically significant with *p* < 0.001. Similar results were also obtained by the author group Del Río-Arteaga et al. [38]. There was no significant difference between the individual groups within the postoperative controls in the present one. This may be due to the fact that the patients had already given a very positive assessment of the postoperative result, after which a further increase in this assessment was hardly possible.

### Conclusions

The fact that revision surgery was not necessary for any of the patients of the considered cohort and in only five of the approx. 620 patients (as of 06/2020) who were subsequently treated with the same prosthesis speaks for the prosthesis and the operative expertise of the surgeon. In addition, this work was able to show that an increase in the expertise of the surgeon can be demonstrated using these radiological and clinical values as parameters.
